# Longitudinal analysis of cell-free mutated *KRAS* and CA 19–9 predicts survival following curative resection of pancreatic cancer

**DOI:** 10.1186/s12885-020-07736-x

**Published:** 2021-01-11

**Authors:** Saskia Hussung, Dilara Akhoundova, Julian Hipp, Marie Follo, Rhena F. U. Klar, Ulrike Philipp, Florian Scherer, Nikolas von Bubnoff, Justus Duyster, Melanie Boerries, Uwe Wittel, Ralph M. Fritsch

**Affiliations:** 1 Department of Medicine I (Hematology, Oncology and Stem Cell Transplantation), Freiburg University Medical Center, Freiburg, Germany; 2 Department of Medical Oncology and Hematology, Zurich University Hospital, Raemistrasse 100, 8091 Zürich, Switzerland; 3 Department of Surgery, Freiburg University Medical Center, Freiburg, Germany; 4 German Cancer Consortium (DKTK), partner site Freiburg, German Cancer Research Center (DKFZ), Heidelberg, Germany; 5 Comprehensive Cancer Center Freiburg (CCCF), Medical Center - University of Freiburg, Faculty of Medicine, University of Freiburg, Freiburg, Germany; 6 Institute of Medical Bioinformatics and Systems Medicine, University Medical Center Freiburg, Faculty of Medicine, University of Freiburg, Freiburg, Germany; 7 German Cancer Research Center (DKFZ), Heidelberg, Germany

**Keywords:** Liquid biopsy, Droplet digital PCR (ddPCR), Cell-free DNA (cfDNA), Circulating *KRAS* (cf*KRAS*^mut^), Prognostic biomarkers, Pancreatic cancer, Molecular monitoring

## Abstract

**Background:**

Novel biomarkers and molecular monitoring tools hold potential to improve outcome for patients following resection of pancreatic ductal adenocarcinoma (PDAC). We hypothesized that the combined longitudinal analysis of mutated cell-free plasma *KRAS* (cf*KRAS*^*mu*t^) and CA 19–9 during adjuvant treatment and follow-up might more accurately predict disease course than hitherto available parameters.

**Methods:**

Between 07/2015 and 10/2018, we collected 134 plasma samples from 25 patients after R0/R1-resection of PDAC during adjuvant chemotherapy and post-treatment surveillance at our institution. Highly sensitive discriminatory multi-target ddPCR assays were employed to screen plasma samples for cf*KRAS*^*mut*^. cf*KRAS*^*mu*t^ and CA 19–9 dynamics were correlated with recurrence-free survival (RFS) and overall survival (OS). Patients were followed-up until 01/2020.

**Results:**

Out of 25 enrolled patients, 76% had undergone R0 resection and 48% of resected PDACs were pN0. 17/25 (68%) of patients underwent adjuvant chemotherapy. Median follow-up was 22.0 months, with 19 out of 25 (76%) patients relapsing during study period. Median RFS was 10.0 months, median OS was 22.0 months. Out of clinicopathologic variables, only postoperative CA 19–9 levels and administration of adjuvant chemotherapy correlated with survival endpoints. cf*KRAS*^*mu*t.^ was detected in 12/25 (48%) of patients, and detection of high levels inversely correlated with survival endpoint. Integration of cf*KRAS*^*mut*^ and CA 19–9 levels outperformed either individual marker. cf*KRAS*^*mut*^ outperformed CA 19–9 as dynamic marker since increase during adjuvant chemotherapy and follow-up was highly predictive of early relapse and poor OS.

**Conclusions:**

Integrated analysis of cf*KRAS*^*mut*^ and CA 19–9 levels is a promising approach for molecular monitoring of patients following resection of PDAC. Larger prospective studies are needed to further develop this approach and dissect each marker’s specific potential.

**Supplementary Information:**

The online version contains supplementary material available at 10.1186/s12885-020-07736-x.

## Background

Despite significant progress in understanding tumor genetics and the molecular mechanisms driving tumor development and resistance to therapy, only minor improvements have been achieved to date in the treatment of patients with pancreatic ductal adenocarcinoma (PDAC). With an average 5-year overall survival (OS) rate of only 10% across all stages, most patients still succumb to their disease, making PDAC one of the most aggressive tumor entities [1–3]. The only potentially curative treatment is surgical resection of early-stage tumors [4, 5]. However, recurrence rates even after R0 resection remain unacceptably high [6–10]. The integration of more efficacious systemic chemotherapy regimens has improved median overall survival [11], yet responses of individual PDACs to chemotherapy are highly heterogeneous and personalization of perioperative therapy is in its infancy [12–18] .

Consequently, the development and validation of novel biomarkers and molecular monitoring tools to predict disease course and assess efficacy of adjuvant chemotherapy are urgently needed. The analysis of tumor-derived cell-free nucleic acids (ctDNA) extracted from the plasma and other body fluids is a promising tool for molecular diagnostics and non-invasive monitoring of cancer patients [19–26]. Up to 95% of PDACs harbor activating hot spot mutations in *KRAS* which are readily detectable in the circulation of PDAC patients [20, 27–29]. We recently described the development and validation of highly sensitive single-target and discriminatory multi-target *KRAS* ddPCR assays for the analysis of cfDNA [30]. These assays allow identification and quantification of mutated *KRAS* directly from circulation without previous knowledge of tumor *KRAS* mutational status, which is not routinely tested for resectable PDACs.

For this study, we hypothesized that longitudinal assessment of cf*KRAS*^*mut*^ following curative resection of PDAC in combination with established protein biomarkers might better identify patients at risk for imminent tumor relapse, indicate failure of adjuvant treatment and ultimately guide treatment according to molecular monitoring. To study the feasibility of this approach, we analyzed plasma samples collected from patients undergoing adjuvant chemotherapy and post-treatment surveillance at our institution in a single-center retrospective biomarker study aiming to identify associations between cf*KRAS*^mut^ and CA 19–9 dynamics and clinical outcome post PDAC resection.

## Methods

### Study design and population

25 patients with resected pancreatic adenocarcinoma (following R0 or R1 curative-intended resection) were included in a retrospective observational single center biomarker study conducted at Freiburg University Medical Center. Local institutional review board (IRB) approved all study procedures (EK48/18). All patients provided written informed consent for sample collection and analysis. 17/25 patients underwent adjuvant chemotherapy (4/17 Gemcitabine, 4/17 FOLFIRINOX, 8/17 Gemcitabine/Capecitabine, 1/17 Gemcitabine/nab-Paclitaxel). Further inclusion criteria were “collection of first sample within 8 weeks after resection”, and “availability of plasma samples for cfDNA extraction”. Key exclusion criteria included “R2 resection, evidence of metastatic disease on pre- or postoperative CT staging, histologies other than adenocarcinoma”. According to UICC/AJCC, R0 resection was defined as microscopic edge-negative resection, in which no microscopic residual tumor remains. R1 resection was defined as a microscopic residual tumor and R2 as macroscopically visible residual tumor. Primary endpoint was detection of cf*KRAS*^mut^ in at least one sample during study period. Secondary endpoints included association between changes in cfKRAS^mut^ and relapse-free survival (RFS) and overall survival (OS). Additionally, clinical, pathologic, treatment- and outcome-related data were analyzed.

### Collection of patient samples and CA 19–9 analysis

Blood samples were collected at a median of 40 days (95% CI 26–50) after resection, prior to adjuvant chemotherapy and during 3-monthly routine clinical follow-up visits. CA 19–9 measurements were performed at our center’s fully certified clinical chemistry facilities. The threshold value for CA 19–9 positivity was 36 U/ml.

### Extraction of cell-free DNA (cfDNA) from plasma samples

Blood samples were collected using commercially available EDTA tubes and plasma was extracted and frozen within one hour of collection. Plasma was extracted through two subsequent centrifugation steps at 3000 rpm and 14,000 rpm, each for 10 min at 4 °C. Obtained plasma was stored at − 80 °C until extraction of cfDNA. cfDNA was extracted from 4 ml plasma following the SEP/SBS protocol of the PME-free circulating DNA extraction kit (Analytik Jena, cat. no. 845-IR-0003050), following manufacturer’s instructions. Two subsequent elution steps with each 30 μl Elution Buffer were performed to optimize the yield of extracted cfDNA. DNA was stored at − 20 °C until cfDNA quantification. cfDNA was evaluated with fragment analyzer and quantified using Qubit 2.0 fluorometer. In patients with resectable PDAC, DNA yield from 4 ml of plasma typically ranged from 1 to 20 ng/μl.

### Droplet digital PCR (ddPCR)

ddPCR for cf*KRAS*^mut^ was performed as recently described [29]. Locked nucleic acid (LNA) probes and corresponding primer pairs for *KRAS* mutations were designed using Beacon Designer v.8.20 software (Premier Biosoft, Palo Alto, California, USA) and manufactured by Integrated DNA Technologies (IDT, Inc., Coralville, Iowa, USA). Wild type (WT) probes were labelled with hexachlorofluorescein (HEX), mutant (MUT) probes with 6-carboxyfluorescein (FAM). Primer and Probe sequences are listed in Supplemental Table 1. Primers, probes, template DNA and nuclease-free water (Ambion, Austin, TX) were added to ddPCR Supermix for Probes (Bio-Rad, cat. no #186–3024). Reaction mix was set up as recommended. 20 μl of this reaction mix along with 70 μl reader oil were transferred into cartridges of a QX100/200TM Droplet Generator (Bio-Rad, cat. no. #1863002) following manufacturer’s instructions. All samples were assayed in quadruplicates. Droplets were generated, transferred into a 96-well PCR plate (Bio-Rad, cat. no. #12001925) and PCR was then run on a C1000 Touch™ Thermal Cycler (Bio-Rad, cat. no. #1851197). Finally, samples were analyzed on a QX100/200TM Droplet Reader (Bio-Rad, cat. no. 1863003) using QuantaSoft v1.7.4.0917 (Bio-Rad, cat.no. #1864011). Internal ddPCR controls were carried out as previously published [29].

The absolute number of copies per milliliter of blood were calculated as follows: Copies/mL plasma = (copies per μL of reaction as per QuantaSoft analysis software version 1.7.4.0917) × (volume of ddPCR reaction) × ([volume eluted/volume of DNA used in reaction]/volume of plasma used for cfDNA extraction). Mutant allele frequency was calculated as: Mutant allele frequency = mutant copies/mL of plasma / (mutant copies/mL of plasma + wild−type copies/mL of plasma).

Limit of detection (LOD) and limit of blank (LOB) of the individual assays have been previously described [30] .

In brief, cfDNA was screened for the presence of the 11 most commonly found *KRAS* hot spot mutations, in PDAC, covering more than 90% of PDAC cases. Highly sensitive single-target assays were used to confirm presence of the mutation identified.

### Statistical analysis

Recurrence-free survival (RFS) was defined as time from resection of PDAC to the first radiologic recurrence (local or distant) or death due to PDAC. Overall survival (OS) was defined as time from the date of diagnosis until death due to any cause. The Kaplan–Meier survival analysis was performed to calculate both RFS and OS. Univariate analyses were performed using the log-rank test. In order to explore independent prognostic factors for RFS and OS, we used backward stepwise Cox regression modeling to estimate hazard ratio (HR) with 95% confidence interval (CI). To compare independent variables, Chi-squared or Fisher’s exact test and the Mann–Whitney (rank-sum) test were performed. All statistical analyses were performed using GraphPad Prism Version 5.03 (GraphPad Software, Inc., La Jolla, California, USA) and SPSS 25 software Version 1.0.0.1327 (IBM Corporation, New York, United States). *P* values < 0.05 were considered as statistically significant.

## Results

### Patient cohort

25 patients with non-metastatic, R0/R1-resected adenocarcinoma of the pancreas were included in the study. Patient characteristics are summarized in Table 1. R0 resection rate was 76% (19/25), 12/25 (48%) of tumors were nodal negative (pN0). 17/25 (68%) patients underwent adjuvant chemotherapy. Median follow-up for the cohort was 22.0 months, with 19 out of 25 (76%) patients relapsing during this period. Median RFS for the cohort was 10.0 months, median OS was 22.0 months. We performed univariate and multivariate survival analyses (Supplemental Tables 2 and 3, Figure S1) for established clinicopathologic variables. We found no significant correlation between R0 vs R1 resection and RFS or OS (Figure S1 A, B). However, we identified a significant inverse correlation between elevated CA 19–9 in the first sample collected after resection and RFS and OS (Figure S1 C, D) and significantly better OS (Figure S1 F) but not RFS (Figure S1 E) for patients undergoing adjuvant chemotherapy.

**Table 1:** Patient and tumor characteristics

Clinicopathologic features	*n* = 25 (%)
Median age (years)	75
Age range	42–81
Sex	
Male	18 (72)
Female	7 (28)
Tumor location	
Pancreas head	20 (80)
Pancreas body & tail	5 (20)
T stage	
T1 – T2	7 (28)
T3	18 (72)
N status	
N0	12 (48)
N1–2	13 (52)
R status	
R0	19 (76)
R1	5 (20)
Rx	1 (4)
Lymphovascular invasion	
L0	15 (60)
L1	10 (40)
Perineural invasion	
Pn0	2 (8)
Pn1	23 (92)
Grading	
G2	13 (52)
G3	12 (48)
Adjuvant chemotherapy	
Yes	17 (68)
No	8 (32)
Time to relapse (months)	
Median	10
Range	0.5–42
Overall survival (months)	
Median	22
Range	0.5–46

### Analysis of plasma cfKRAS^mut^

We analyzed 134 plasma samples collected from 25 patients at routine follow-ups before, during and after adjuvant chemotherapy. First samples were taken at a median of 40 days (95% CI 26–50) after resection prior to adjuvant chemotherapy. Median number of samples collected was 4 samples per patient (95% CI 3–5 samples). Median time interval between sampling was 70 days (95% CI 63–91). We screened cfDNA extracted from plasma samples for the presence of cfKRAS^*mut*^ with recently described discriminatory multi-target *KRAS* ddPCR assays, covering the 11 most common *KRAS* hot spot mutations in PDAC [30]. At the postoperative stage, no molecular pathology data was available for any tumor. However, for a subset of patients, *KRAS* mutational status became available at relapse (Supplemental Table 4).

Across all samples analyzed, cf*KRAS*^*mu*t^ was detected in 34/134 (25%) samples and 12/25 (48%) of patients for at least one time point. In 14/15 (93.33%) patients with later on determined tumor tissue *KRAS* mutational status, the SNV detected by ddPCR in plasma (cf*KRAS*^mut^) at any time point matched the *KRAS* SNV detected in tissue analysis (Supplemental Table 4), confirming the validity of ddPCR cf*KRAS*^mut^ analysis. In 0/134 (0%) of plasma samples, a concurrent second *KRAS* SNV could be detected above assay threshold.

### Association of cfKRAS^mut^ and elevated CA 19–9 levels with survival endpoints

**Fig. 1:**
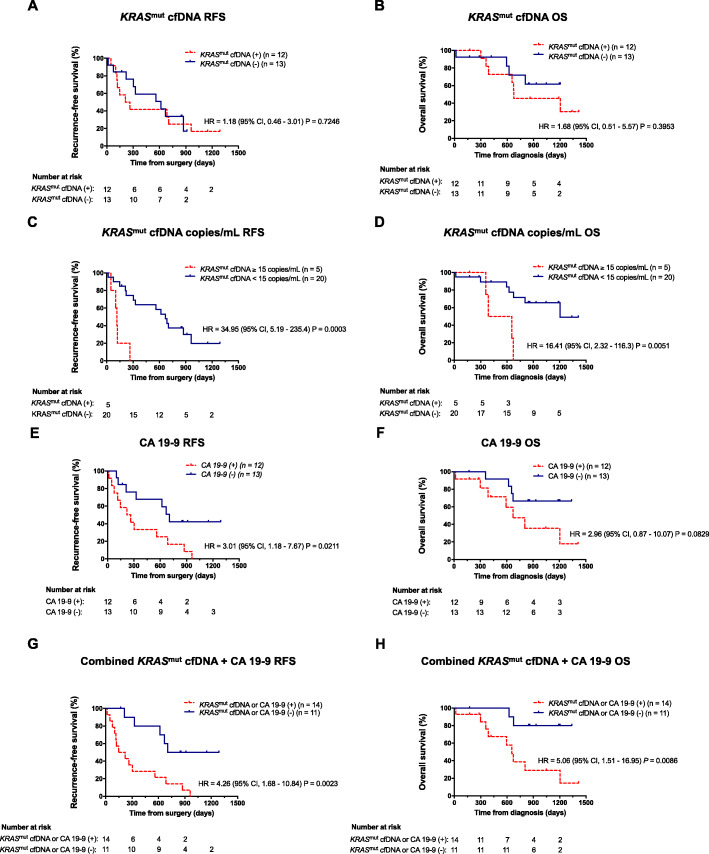
*Association of cfKRAS*^*mut*^ *detection and elevated CA 19–9 levels with survival endpoints.* **a**, **b** Kaplan-Meier estimates of RFS (**a**) and OS (**b**) for patients following curative resection of PDAC with versus without detectable cf*KRAS*^mut^ at any time point during study period. **c**, **d**. A more stringent cf*KRAS*^mut^ cut-off level of > 15 copies/mL plasma was chosen. **e**, **f** Kaplan-Meier estimates of RFS (**e**) and OS (**f**) for resected PDAC patients with elevated (> 36 U/mL) versus normal (≤ 36 U/mL) CA 19–9 levels at any time point during observation period. **g**, **h** Kaplan-Meier estimates of RFS (**g**) and OS (**h**) for resected PDAC patients with either CA 19–9 positivity or cf*KRAS*^mut^ levels > 15 copies/mL cfKRAS during study course. OS, overall survival; RFS, recurrence-free survival; PDAC, pancreatic ductal adenocarcinoma

Detection of cf*KRAS*^mut^ at any time point during study course above assay threshold was not associated with RFS or OS (Fig. 1a, b). However, post-hoc analysis uncovered that a more stringent cut-off level of 15 copies *KRAS*^mut^ per ml plasma for cf*KRAS*^mut^ detected at any time point during study period was strongly associated with early relapse and poor survival (Fig. 1c, d). Analogously, when analyzing *KRAS* variant allele frequencies (VAF) instead of DNA copy numbers, detection of cf*KRAS*^mut^ above a threshold of 0.5% VAF, as determined by post hoc analysis, was associated with inferior RFS and OS (Supplemental Figure 2 A, B). Notably, all 5/25 patients with a copy number of 15 copies *KRAS*^mut^ per ml or higher also had a VAF above 0.5%, while 18/20 patients with a copy number < 15 copies *KRAS*^mut^ per ml had a VAF < 0.5%, suggesting large overlap between the two distinct ways of analysis. CA 19–9 levels were determined from the same blood collections. 12/25 (48%) of patients had at least one blood sample with CA 19–9 above normal range during study course. Increased CA 19–9 at any time point was associated with significantly inferior RFS and a non-significant trend towards inferior OS (Fig. 1e, f). Notably, only 6/12 (50%) patients were double positive for cf*KRAS*^mut^ and CA 19–9, indicating that cf*KRAS*^mut^ and CA 19–9 positivity are not redundant. Patients with either CA 19–9 positivity or cf*KRAS*^mut^ levels > 15 copies/mL during study course (14/25, 56%) showed inferior RFS and OS, indicating that the integration of both biomarkers might be predictive and prognostic for a larger group of patients than assaying them individually (Fig. 1g, h). Survival of double positive patients was similar to single positive patients in our cohort (data not shown). When analyzing associations between liquid biomarkers and clinicopathologic variables, there was no significant correlation between R status and postoperative levels of CA 19–9, cf*KRAS*^mut^ or total cell-free DNA (cfDNA) concentrations (Supplemental Figure 2 C, D, E).

### Association of cfKRAS^mut^ and CA 19–9 dynamics with survival

**Fig. 2:**
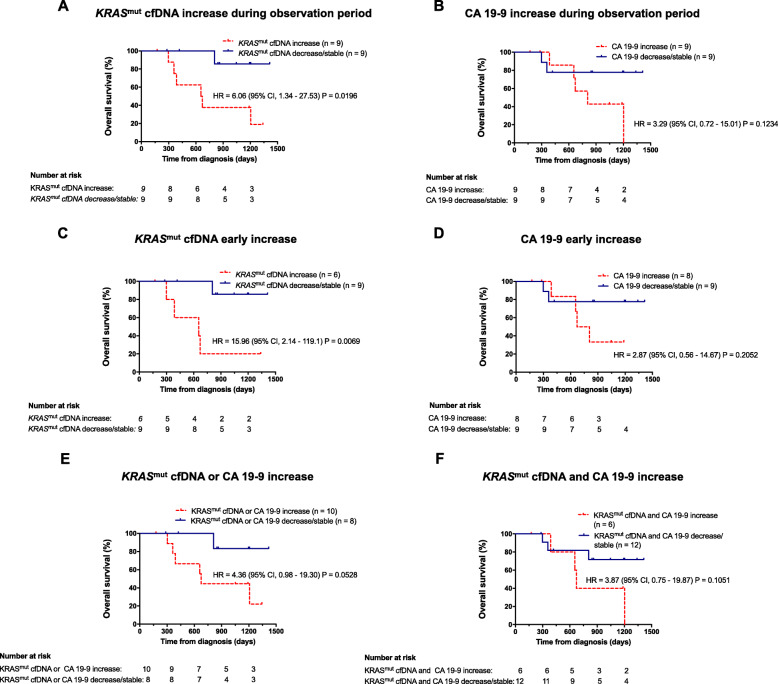
*Association of cfKRAS*^*mut*^ *and CA 19–9 dynamic changes with survival endpoints.* **a**, **b** Kaplan-Meier estimates of OS for resected PDAC patients with increase of cf*KRAS*^mut^ (**a**) or CA 19–9 (**b**) during observation period. **c**, **d** Kaplan-Meier estimates of OS for resected PDAC patients with early increase of cf*KRAS*^mut^ (**a**) or CA 19–9 (**b**) during observation period. Increase of cf*KRAS*^mut^ or CA 19–9 during observation period was defined as numerical increase of the respective parameter in initially positive patients or rise above threshold in initially negative patients. Early increase was defined as increase within 6 months after surgery. **e** Kaplan-Meier estimates of OS for resected PDAC patients with combined early increase of cf*KRAS*^mut^ or CA 19–9. **f** Kaplan-Meier estimates of OS for resected PDAC patients with combined early increase of cf*KRAS*^mut^ and CA 19–9. OS, overall survival; RFS, recurrence-free survival; PDAC, pancreatic ductal adenocarcinoma

Protein tumor markers and cfDNA are highly dynamic biomarkers for the molecular monitoring of disease course and treatment response. We therefore next analyzed whether changes over time in either biomarker are associated with outcome in our cohort. For each 9/18 (50%) patients with a sufficient number of follow-up samples, cf*KRAS*^mut^ or CA 19–9 levels increased during observation period. Increase of cf*KRAS*^mut^ or CA 19–9 during observation period was defined as numerical increase of the respective parameter in initially positive patients or rise above threshold in initially negative patients. Increase of cf*KRAS*^mut^ was associated with significantly reduced OS (Fig. 2a), while increase of CA 19–9 was not significantly associated inferior OS (Fig. 2b). Similarly, early increase of cf*KRAS*^mut^, defined as increase within 6 months after surgery, was strongly associated with inferior OS while early CA 19–9 increase was not significantly associated with shorter OS (Fig. 2c, d). Integrating both markers for the analysis of dynamic changes over time did not outperform cf*KRAS*^mut^ alone (Fig. 2e, f), suggesting that cf*KRAS*^mut^ might be the biomarker of choice for longitudinal monitoring in this setting.

### Single patient analysis

**Fig. 3:**
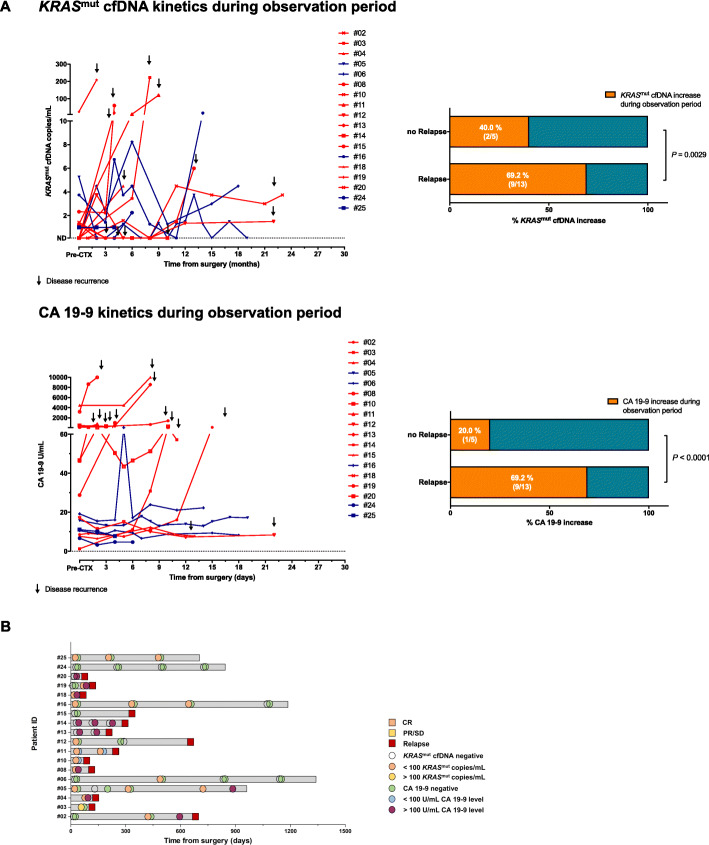
Longitudinal cf*KRAS*^mut^ and CA 19–9 monitoring. **a** *Top left*: Absolut levels of cf*KRAS*^mut^ during observation period. Patients with relapse during study period are marked red. Black arrow mark the time of disease recurrence. *Top right:* Relapse versus non-relapse patients with increase in cf*KRAS*^mut^ during observation period. Fisher’s exact test was used to test for statistical significance between the two groups. *P* values < 0.05 were considered significant. *Bottom left*: Absolut levels of CA 19–9 during observation period. Patients with relapse are marked red. Black arrows mark the time of disease recurrence. *Bottom right:* Relapse versus non-relapse patients with increase in cf*KRAS*^mut^ during observation period. Fisher’s exact test was used to test for statistical significance between the two groups. *P* values < 0.05 were considered significant. **b** Swimmers plot of disease course of resected PDAC patients. cf*KRAS*^mut^ and CA 19–9 analysis in blood were compared to clinical course of disease before and during adjuvant chemotherapy. CR, complete response; PD, progressive disease; PR, partial response; SD, stable disease

Figure 3 illustrates the relationship between cf*KRAS*^mut^ and CA 19–9 (Fig. 3a) dynamics and tumor relapse for individual patients. 13/18 patients in the analysis relapsed during observation period. Increase of cf*KRAS*^mut^ or CA 19–9 was significantly associated with relapse. 09/13 patients with relapse during observation period showed an increase in either CA 19–9 or cf*KRAS*^mut^ (Fig. 3a), 6 out of these 9 patients showed an increase for both markers indicating partial overlap (not shown). Notably, 2/5 patients with no relapse during observation period still showed an increase in either CA 19–9 or cf*KRAS*^mut^ suggesting either insufficient duration of follow-up or suboptimal specificity when integrating both markers for analysis. Single-patient analysis also illustrates dynamic changes during adjuvant chemotherapy and follow-up with several patients showing transient increases followed by decreases of either marker. Figure 3b illustrates that, in most patients, relapsed was proceeded by a strong increase of CA 19–9 or cf*KRAS*^mut^. However, single patient analyses also suggests that both cf*KRAS*^mut^ and CA 19–9 are highly dynamic biomarkers and that individual patterns can be highly heterogeneous requiring well-defined cut-off levels and extensive clinical validation for future clinical application.

## Discussion

In an exploratory analysis, we followed a small cohort of pancreatic cancer patients after curative resection of pancreatic adenocarcinoma through adjuvant therapy and post-treatment follow-up. We analyzed mutated *KRAS* in cell-free DNA with discriminatory ddPCR assays and integrated results with CA 19–9 levels for association with relapse and survival endpoints. Numerous studies have unveiled the potential of the analysis of cell-free mutated tumor DNA as novel diagnostic [27], predictive [31–33] and prognostic [31, 32, 34–37] biomarker for pancreatic cancer.

What takes our study apart is the use of discriminatory multi-target *KRAS* ddPCR assays [30] to directly identify *KRAS* SNVs without performing previous tumor NGS. These assays have higher sensitivity compared to many available NGS-based assays [29]. Moreover, the input volume of 4 ml plasma for cfDNA extraction might have contributed to the comparably higher sensitivity of our assays compared to previous reports [26]. In comparison to more sophisticated NGS panels specifically developed for cfDNA analysis [29, 38], multi-target ddPCR assays are associated with much lower assay costs, allowing for the serial analysis through clinical course analogous to CA 19–9 levels. Using these assays, our cf*KRAS*^mut^ detection rate in the cohort was similar to other published data for patients following PDAC resection [20, 31, 36]. A very high concordance rate between tumor tissue und detected cfDNA *KRAS* SNVs further validates our approach.

In our cohort, detection of cf*KRAS*^mut^ in the first postoperative sample alone did not significantly correlate with survival (data not shown), while elevated CA 19–9 levels at first presentation were associated with poor outcome. Similarly, positivity for cf*KRAS*^mut^ at any time point above assay threshold alone was not significantly associated with survival. However, when choosing a more stringent cf*KRAS*^mut^ cut-off or when analyzing dynamic changes (increase vs non-increase), cf*KRAS*^mut^ was strongly associated with survival and outperformed CA 19–9 levels for association with relapse and OS, highlighting the importance of identifying clinically validated cut-offs for cfDNA analysis [39–41] and also underlining the limitations associated with analyzing a small patient cohort.

One main finding of our analysis was that cf*KRAS*^mut^ positivity and CA 19–9 elevation are only partially overlapping and that combining both parameters identifies a larger cohort of patient with poor outcome. Several studies have suggested integration of established and experimental protein biomarkers with cfDNA analysis for pancreatic cancer early diagnostics [27, 28, 42–44], identification of minimal residual disease [45] and molecular monitoring for advanced disease [26, 41]. Our approach is focused on clinical applicability and feasibility through integration of the two relatively easy-to-assess biomarkers cf*KRAS*^mut^ analysis and CA 19–9. Notably, a recent large multi-center case-control study did not find a benefit if cf*KRAS*^mut^ analysis as compared to CA 19–9 analysis in pancreatic cancer patients across all stages of disease [26]. In this study, however, reported detection rates of cf*KRAS*^mut^ were overall lower than in our study, probably due to differences in assay technology. Our data suggest that CA 19–9 and cf*KRAS*^mut^ levels each have their own distinct advantages and disadvantages and that integrating them for analysis might be superior to analyzing them individually, the question of how best to integrate both biomarkers for clinical practice remains challenging, which is also illustrated by the analysis of single patient’s disease course in our cohort.

A major limitation of our study is the small cohort size, which makes it difficult to define clinically relevant cut-off levels forcf*KRAS*^mut^ and to optimize integration of CA 19–9 with cf*KRAS*^mut^. However, despite these limitations, our study also points to the potential of clinically further developing cf*KRAS*^mut^ as prognostic and predictive biomarkers for the management of resectable PDAC. Most importantly, further studies will have to explore the potential of biomarker-based therapeutic intervention for pancreatic cancer. Systemic treatment options for PDAC are limited to a small number of combination chemotherapy regimens [46, 47] and some recent developments in personalized treatment based on molecular profiling [14]. A switch of adjuvant chemotherapy regimen based on molecular monitoring appears feasible yet will need extensive clinical validation in interventional trials, especially since established adjuvant treatment standards took so many years to establish.

In summary our study proposed a clinically feasible approach to assay cf*KRAS*^mut^ together with CA 19–9 in patients following curative resection of PDAC. Through combination of both markers, patients could be better stratified in terms of relapse risk and overall prognosis.

## Conclusion

The integrated longitudinal analysis of cf*KRAS*^*mut*^ and CA 19–9 levels holds potential for the molecular monitoring of patients following resection of PDAC. Larger cohorts and prospective trials are required to establish clinically relevant cut-off levels and to better unravel the relationships between biomarker dynamics and clinical relapse.

## Supplementary Information

**Additional file 1: Supplemental Figure 1.** Univariate survival analyses for established clinicopathologic variables. A, B) Kaplan-Meier estimates of RFS (A) and OS (B) for PDAC patients stratified in two groups: patients with R1 vs. R0 resection. (C, D) Kaplan-Meier estimates of RFS (C) and OS (D) for PDAC patients stratified by postoperative CA 19–9 levels: elevated (> 36 U/mL) versus normal (≤ 36 U/mL). (E, F) Kaplan-Meier estimates of RFS (E) and OS (F) for resected PDAC patients with/without adjuvant chemotherapy. OS, overall survival; RFS, recurrence-free survival; PDAC, pancreatic ductal adenocarcinoma. Univariate analyses were performed using the log-rank test. **Supplemental Figure 2.** Association of cfKRAS^mut^ with survival endpoints and clinicopathologic variables. (A, B) Kaplan-Meier estimates of RFS (A) and OS (B) for patients following curative resection of PDAC with versus without cf*KRAS*^mut^ cut-off level of > 0.5% variant allele frequency (VAF) at any time point during study period. (C, D, E) cf*KRAS*^mut^ (C), total cell-free DNA (D) and CA 19–9 levels (E) in untreated resected PDAC patients were compared to resection margin. OS, overall survival; RFS, recurrence-free survival; PDAC, pancreatic ductal adenocarcinoma

**Additional file 2: Table S1.** Primer and probe sequences for KRAS ddPCR assays. **Table S2.** Overall survival analysis by clinico-pathologic variables. **Table S3.** Recurrence-free survival analysis by clinico-pathologic variables. **Table S4.** Comparison between tissue analyses and cfDNA ddPCR.

## Data Availability

The datasets used and/or analyzed during the current study are available from the corresponding author on reasonable request.

## Abbreviations

cfDNA: Cell-free DNA
cf*KRAS*^*mu*t^: Mutated cell-free plasma *KRAS*
ctDNA: Tumor-derived cell-free nucleic acids
ddPCR: Droplet digital PCR
IDT: Integrated DNA Technologies
IRB: Institutional review board
HR: Hazard ratio
LOB: Limit of blank
LOD: Limit of detection
mFOLFIRINOX: 5-fluorouracil, irinotecan, oxaliplatin, leucovorin
NGS: Next generation sequencing
OS: Overall survival
PCR: Polymerase chain reaction
PDAC: Pancreatic ductal adenocarcinoma
RFS: Recurrence-free survival
SNV: Single nucleotide variants
