# Supplementary material for: Longitudinal analysis of cell-free mutated KRAS and CA 19–9 predicts survival following curative resection of pancreatic cancer
Source: BMC Cancer. 2021 Jan 11;21:49. doi: 10.1186/s12885-020-07736-x (PMC7802224; doi:10.1186/s12885-020-07736-x)
Supplement: Supplementary file 1 — Additional file 1: Supplemental Figure 1. Univariate survival analyses for established clinicopathologic variables. A, B) Kaplan-Meier estimates of RFS (A) and OS (B) for PDAC patients stratified in two groups: patients with R1 vs. R0 resection. (C, D) Kaplan-Meier estimates of RFS (C) and OS (D) for PDAC patients stratified by postoperative CA 19–9 levels: elevated (> 36 U/mL) versus normal (≤ 36 U/mL). (E, F) Kaplan-Meier estimates of RFS (E) and OS (F) for resected PDAC patients with/without adjuvant chemotherapy. OS, overall survival; RFS, recurrence-free survival; PDAC, pancreatic ductal adenocarcinoma. Univariate analyses were performed using the log-rank test. Supplemental Figure 2. Association of cfKRASmut with survival endpoints and clinicopathologic variables. (A, B) Kaplan-Meier estimates of RFS (A) and OS (B) for patients following curative resection of PDAC with versus without cfKRASmut cut-off level of > 0.5% variant allele frequency (VAF) at any time point during study period. (C, D, E) cfKRASmut (C), total cell-free DNA (D) and CA 19–9 levels (E) in untreated resected PDAC patients were compared to resection margin. OS, overall survival; RFS, recurrence-free survival; PDAC, pancreatic ductal adenocarcinoma [file 12885_2020_7736_MOESM1_ESM.zip › Supplemental Figure legend revision 22-10-20R3.docx]

**Supplemental Figure 1.** Univariate survival analyses for established clinicopathologic variables

A, B) Kaplan-Meier estimates of RFS (A) and OS (B) for PDAC patients stratified in two groups: patients with R1 vs. R0 resection. (C, D) Kaplan-Meier estimates of RFS (C) and OS (D) for PDAC patients stratified by postoperative CA 19-9 levels: elevated (> 36 U/mL) versus normal (≤ 36 U/mL). (E, F) Kaplan-Meier estimates of RFS (E) and OS (F) for resected PDAC patients with/without adjuvant chemotherapy. OS, overall survival; RFS, recurrence-free survival; PDAC, pancreatic ductal adenocarcinoma. Univariate analyses were performed using the log-rank test.

**Supplemental Figure 2.** Association of cfKRAS^mut^ with survival endpoints and clinicopathologic variables

(A, B) Kaplan-Meier estimates of RFS (A) and OS (B) for patients following curative resection of PDAC with versus without cf*KRAS*^mut^ cut-off level of > 0.5 % variant allele frequency (VAF) at any time point during study period. (C, D, E) cf*KRAS*^mut^ (C), total cell-free DNA (D) and CA 19-9 levels (E) in untreated resected PDAC patients were compared to resection margin. OS, overall survival; RFS, recurrence-free survival; PDAC, pancreatic ductal adenocarcinoma
