# Supplementary material for: Longitudinal analysis of cell-free mutated KRAS and CA 19–9 predicts survival following curative resection of pancreatic cancer
Source: BMC Cancer. 2021 Jan 11;21:49. doi: 10.1186/s12885-020-07736-x (PMC7802224; doi:10.1186/s12885-020-07736-x)
Supplement: Supplementary file 2 — Additional file 2: Table S1. Primer and probe sequences for KRAS ddPCR assays. Table S2. Overall survival analysis by clinico-pathologic variables. Table S3. Recurrence-free survival analysis by clinico-pathologic variables. Table S4. Comparison between tissue analyses and cfDNA ddPCR. [file 12885_2020_7736_MOESM2_ESM.docx]

**Table S1. Primer and probe sequences for *KRAS* ddPCR assays**

| Primers | | | |
| --- | --- | --- | --- |
| Gene | Target | Direction | Sequence |
| *KRAS* | G12/G13 | forward | 5'- GCC TGC TGA AAA TGA CTG -3' |
|  |  | reverse | 5'- GCT GTA TCG TCA AGG CAC -3' |
|  | G12A&C | forward | 5'- GGA TCA TAT TCG TCC ACA A -3' |
|  |  | reverse | 5'- CCT GCT GAA AAT GAC TGA A -3' |
|  | Q61 | forward | 5'- AGT CCT CAT GTA CTG GTC -3' |
|  |  | reverse | 5'- CCT GTC TCT TGG ATA TTC TC -3' |
|  | Q61R | forward | 5'- TGG CAA ATA CAC AAA GAA AG -3' |
|  |  | reverse | 5'- CCT GTC TCT TGG ATA TTC TC -3' |
| Probes | | | |
| Gene | Target | Mutation | Sequence |
| *KRAS* | G12 | wild type | 5'- /5HEX/CC+A +C+C+A G+CT C/3IABkFQ/ -3' |
|  |  | c.35G>T | 5'- /56-FAM/CG+C C+A+A +CAG +CT/3IABkFQ/ -3' |
|  |  | c.35G>A | 5'- /56-FAM/CCA +C+G+A +GC+T C/3IABkFQ/ -3' |
|  |  | c.34G>C | 5'- /56-FAM/CGC +C+A+T +CA+G C/3IABkFQ/ -3' |
|  |  | c.35G>C | 5'- /56-FAM/AGC +TG+C T+GG +CGT A/3IABkFQ/ -3' |
|  |  | c.34G>T | 5'- /56-FAM/TAC +GC+C A+CA +AGC TC/3IABkFQ/ -3' |
|  |  | c.34G>A | 5'- /56-FAM/TAC G+CC +AC+T A+GC TCC A/3IABkFQ/ -3' |
|  | G13 | wild type | 5'- /5HEX/CTG G+TG +GC+G T+AG GCA A/3IABkFQ/ -3' |
|  |  | c.38G>A | 5'- /56-FAM/CTG G+TG +AC+G T+AG GCA A/3IABkFQ/ -3' |
|  | Q61 | Wild type | 5'- /5HEX/TAC TCC +TCT +TGA +CCT +GCT G/3IABkFQ/ -3' |
|  |  | c.183A>C | 5'- /56-FAM/TAC TC+C TC+G TG+A CC+T GCT /3IABkFQ/ -3' |
|  |  | c.182A>T | 5'- /56-FAM/TAC TCC +TCT +AGA +CCT +GCT G/3IABkFQ/ -3' |
|  |  | c.182A>G | 5'- /56-FAM/ACT C+CT +CT+C G+AC CTG C/3IABkFQ/ -3' |
|  |  | c.181C>A | 5'- /56-FAM/TAC T+CC T+CT T+TA C+CT GCT /3IABkFQ/ -3' |

**Table S2.** **Overall survival analysis by clinico-pathologic variables**

| **Variable** | **Univariate analysis** | | |  | **Multivariate analysis** | | |
| --- | --- | --- | --- | --- | --- | --- | --- |
|  | **HR** | **95% CI** | ***P*** |  | **HR** | **95% CI** | ***P*** |
| Age  < median versus ≥ median^$^ | 2.76 | 0.787-9.689 | 0.1129 |  |  |  |  |
| Gender  Male versus female | 0.29 | 0.061-1.343 | 0.1128 |  |  |  |  |
| Tumor location pancreas  Head versus body & tail | 0.96 | 0.250-3.674 | 0.9495 |  |  |  |  |
| Tumor size  ≤ 2cm versus > 2cm | 0.88 | 0.098-7.956 | 0.9106 |  |  |  |  |
| Tumor stage  T1-T2 versus T3 | 1.76 | 0.483-6.392 | 0.3923 |  |  |  |  |
| Lymph node status  N0 versus N1/2 | 0.38 | 0.114-1.292 | 0.1219 |  |  |  |  |
| Lymphovascular invasion  Present versus absent | 1.20 | 0.360-4.006 | 0.7651 |  |  |  |  |
| Resection margin status  R0 versus R1 | 1.18 | 0.230-6.000 | 0.8455 |  |  |  |  |
| Tumor differentiation  Well/medium versus poor | 1.68 | 0.500-5.615 | 0.4028 |  |  |  |  |
| CA 19-9 status post-OP^§^  ≤ 36 versus > 36 U/mL | 5.34 | 1.158-24.57 | **0.0317** |  | 4.76 | 1.310-17.285 | **0.018** |
| Adjuvant Chemotherapy  Yes versus no | 7.76 | 1.785-33.70 | **0.0063** |  |  |  |  |

**Table S3. Recurrence-free survival analysis by clinico-pathologic variables**

| **Variable** | **Univariate analysis** | | |  | **Multivariate analysis** | | |
| --- | --- | --- | --- | --- | --- | --- | --- |
|  | **HR** | **95% CI** | ***P*** |  | **HR** | **95% CI** | ***P*** |
| Age  < median versus ≥ median | 1.84 | 0.730-4.627 | 0.1972 |  |  |  |  |
| Gender  Male versus female | 0.76 | 0.252-3.323 | 0.6361 |  |  |  |  |
| Tumor location pancreas  Head versus body & tail | 1.32 | 0.424-4.124 | 0.6301 |  |  |  |  |
| Tumor size  ≤ 2 cm versus > 2 cm | 1.64 | 0.330-8.161 | 0.5444 |  |  |  |  |
| Tumor stage  T1-T2 versus T3 | 1.32 | 0.464-3.737 | 0.6056 |  |  |  |  |
| Lymph node status  N0 versus N1/2 | 0.71 | 0.281-1.791 | 0.4668 |  |  |  |  |
| Lymphovascular invasion  Present versus absent | 1.53 | 0.601-3.908 | 0.3715 |  |  |  |  |
| Resection margin status  R0 versus R1 | 2.33 | 0.656-8.276 | 0.1907 |  |  |  |  |
| Tumor differentiation  Well/medium versus poor | 1.82 | 0.731-4.540 | 0.1978 |  |  |  |  |
| CA 19-9 status post-OP^§^  ≤ 36 versus > 36 U/mL | 4.72 | 1.534-14.51 | **0.0068** |  | 4.25 | 1.610-11.220 | **0.003** |
| Adjuvant Chemotherapy  Yes versus no | 2.17 | 0.730-6.444 | 0.1632 |  |  |  |  |

^$^Median Age = 75; ^§^Median Time post-OP = 40 days

**Table S4.**

| **Sample No.** | **KRAS genotype** | **SNV^$^** | **Tissue**  **analysis** | **Plasma**  **(ddPCR)** |
| --- | --- | --- | --- | --- |
| #02 | G12R | c.34G>C | + | + |
| #03 | G12D | c.35G>A | + | + |
| #05 | G12D | c.35G>A | + | + |
| #06 | G12D | c.35G>A | + | + |
| #07 | G12D | c.35G>A | + | - |
| #10 | G12D | c.35G>A | + | + |
| #11 | G12D | c.35G>A | + | + |
| #14 | WT^§^ | / | - | - |
| #15 | G12D | c.35G>A | + | + |
| #16 | G12R | c.34G>C | + | + |
| #17 | G12V | c.35G>T | + | + |
| #19 | Q61R | c.182A>G | + | + |
| #20 | G12V | c.35G>T | + | + |
| #24 | G12V | c.35G>T | + | + |
| #25 | G12D | c.35G>A | + | + |

^$^SNV, single nucleotide variant; ^§^WT, Wild-type

**Table S4. Comparison between tissue analyses and cfDNA ddPCR**.

cfDNA was extracted from plasma of PDAC patients with at this point unknown *KRAS* mutational status. *KRAS* plasma SNV were identified with ddPCR assays. For the subset of patients listed here, tissue analyses were performed later during routine pathology workup and results are displayed for comparison.
